# Nootropic activity of methanolic extract from Evolvulus alsinoides Linn. in mice with scopolamine-induced amnesia

**DOI:** 10.6026/973206300191212

**Published:** 2023-12-31

**Authors:** Karnam Nithya, Rajaram Siddaraman, PP Sheelajoice, Mani Rajarathinam, Vurimi Bhopal Chandra

**Affiliations:** 1 Department of Pharmacology, Vinayaka Missions University, Salem (Deemed to be university), Chinna Seeragapadi, Salem - 636308, Tamilnadu, India; 2 Department of Pharmacology, VMKV Medical College, Chinna Seeragapadi, Salem - 636308, Tamil Nadu, India; 3 Department of Physiology, VMKV Medical College, Chinna Seeragapadi, Salem - 636 308, Tamil Nadu, India; 4 Department of Pharmacology, GMKMC Medical College, Shavapet Salem 636002, Tamil Nadu, India; 5 Department of Pharmacology, Narayana Medical College, Nellore - 524 003, AP, India

**Keywords:** Nootropic activity, methanolic extract, Evolvulus alsinoides Linn, vishnukranthi, scopolamine

## Abstract

Plants have been used as therapeutic agents in both un-ionized (Unani, Ayurveda) and unstructured forms since ancient times. Therefore, it is of interest to document the nootropic activity of methanolic extract from Evolvulus alsinoides Linn (Vishnukranthi) in mice with scopolamine-induced amnesia. Healthy male Swiss albino mice ranging between 25 and 30 g were used in the study. Scopolamine induced amnesia, the following two tests are performed, elevated plus maze test, passive avoidance test. The mean time spent in the open arm, closed arm, and central platform for each group of animals. The total transitions were 12.6±0.89 by GS group mice, 3.4±0.55 by GSP group mice, 7±0.71 by GSLD group mice and 10±0.71 by GSHD group mice. A significant difference was seen between GS and GSP group mice means. The mean time in the safe zone and shock zone for each group of animals when comparing to Group 2 Vs Group 3, 4 showed a statistical significance of p < 0.05. The findings of this study suggest that Evolvulus alsinoides may be a promising candidate for the development of new treatments for memory impairment and other cognitive disorders. It should be noted that more data is needed to confirm the safety and efficacy of Evolvulus alsinoides in humans and to investigate its long-term effects.

## Background:

After recognizing the harmful effects and limitations of synthetic medications, the world is turning back to traditional systems of medicine. Plants have been used as therapeutic agents in both un-ionized (Unani, Ayurveda) and unstructured forms since ancient times. Vishnukranthi (Evolvulus alsinoides Linn.) is one such powerful herb that has been used by physicians for centuries [1]. In Unani literature, it is considered a memory enhancer and has been used as a rejuvenator, anti-aging agent, stimulant, and sedative. In Ayurveda, all parts of Evolvulus Alsinoides are used for therapeutic purposes [2]. The medicinal potential of Ayurvedic herbs is vast, and there is a growing interest in exploring this potential. Plant-based medicine is gaining popularity due to the emergence of new techniques for chemical characterization and medical research. Since ancient times, plants have been valued for their ability to heal and relieve pain. Medicinal plants are often used for their healing properties [3]. Many types of herbs are used in traditional folk medicine, and they have a long history of being effective in traditional remedies. In vitro screening methods are also important for identifying promising plant extracts with promising and useful properties for future chemical and medical analysis [4]. Evolvulus alsinoides is a low-growing perennial herb with a slightly branching woody rootstock. It has square, hairy stems that are annual and up to 30 cm long. The branches are often prostrate. Evolvulus alsinoides has small, elliptic leaves that are acute, sensitive, and densely hairy. All parts of this plant are used in Ayurvedic medicine to treat cough, cold, and fever. It is also used to treat inflammation-related neurodegenerative disorders, such as Alzheimer's disease and Parkinson's disease [5]. Evolvulus Alsinoides has been shown to have azoospermic, inotropic, and anti-inflammatory properties. It also has anti-hemorrhagic and antioxidant properties. This herb was used in ancient medicine as a brain tonic, and recent preclinical studies have shown that it is effective in treating neurological disorders such as bronchial asthma and memory loss [6]. The most remarkable property of Evolvulus Alsinoides is its ability to enhance memory and intellect. It has been shown to improve cognitive function in both animals and humans. Evolvulus Alsinoides is a promising herb for the treatment of neurological disorders, and more research is needed to investigate its full potential [7,8]. Evolvulus Alsinoides is a versatile herb with a wide range of medicinal properties. It has been used for centuries in Ayurvedic medicine to treat a variety of conditions, and recent research has shown that it is also effective in treating neurological disorders. Evolvulus Alsinoides is a promising herb for the future of healthcare [9,10]. Taxonomy; Kingdom - Plantae, Phylum - Tracheophytes, Class - Angiospermae Category - Eudicots, Order - Solanales, Family - Convolvulaceae, Genus - Evolvulus, Species - Evolvulus alsinoides [11,12]. Therefore, it is of interest to document the nootropic activity of methanolic extract from Evolvulus Alsinoides linn (Vishnukranthi) in mice with scopolamine-induced amnesia.

## Materials and Methods:

## Plant materials:

The Evolvulus alsinoides plant was collected in July 2022 from Sri Venkateswara University, Chittoor District, Andhra Pradesh, India, from a single herb. Dr K. Madhava Chetty, Plant taxonomist, (IAAT:337), Assistant Professor, Department of Botany, Tirupathi, Andhra Pradesh, India, identified and authenticated the whole plant. Voucher number: 0669 Botanical names Evolvulus alsinoides L., The whole plant was cleaned with fresh running tap water followed by distilled water and dried in a shaded sunlight area after authentication, which was later finely powdered. The powdered plant was subjected to alcoholic extraction by maceration.

## Experimental animals:

Healthy male Swiss albino mice ranging between 25 and 30 g were used in the study. They were kept under (24-270C) room temperature and 60-65 percent humidity) conditions with a light & dark period for 12 hours. Ad libitum, food was available in the form of dried pellets & water as per CPCSEA guidelines. The experimental study got approval from the institutional animal ethical committee (Reg.No.VI/ IAEC/ Dr MGR2053/ PO/ ReBi/ S/ 19/ CPCSEA/ 28.01.2023/ 03).

## Memory and learning activity (Nootropic Activity):

## Animal required:

a) Species: Adult Swiss Albino Mice.

b) Weight: 25-30g

c) Gender: MALE

d) Numbers to be used: 20

e) Number of days each animal will be housed: 30 days

## Animal usage:

The study has two models; scopolamine-induced amnesia 20 mice are used for the study.

## Preparation and mode of administration of drugs:

All drug solutions are freshly prepared.

## Experimental design:

The animals were divided into 4 groups of 5 mice each. The drugs were administered as shown below: 20 mice are used.

## Scopolamine induced amnesia:

Healthy Albino mice of either sex were divided into 4 groups. Each group containing 5 animals and treated for 14 days as follows:(Table 1)

Group 1: Standard (Piracetam 50 mg/kg p.o,) + scopolamine (i.p.),

Group 2: Positive control- Nootropil (scopolamine 1mg/kg [i.p]),

Groups 3 and 4 test groups which receive EA extract, 200 mg and 400 mg/kg, p.o, + scopolamine respectively.

## Exteroceptive behavioural model:

## Elevated plus maze test:

The elevated plus maze served as the exteroceptive behavioural model (where stimulus existed outside the body) to evaluate learning and memory in mice (Joshi *et al.*) [13]. the apparatus comprises two open arms (16 cm x 5 cm) and two covered arms (16 cm x 5 cm x 12 cm). The arms extend from a central platform (5 cm x 5 cm), and the maze is elevated to 25 cm from the floor. On the first day, each mouse was placed at the end of an open arm, facing away from the central platform. Transfer latency (TL) was taken as the time the mouse took to move into one of the covered arms with all four legs. TL was recorded on the first day. If the animal did not enter into one of the covered arms within 90 sec, it was gently pushed into one of the two covered arms and the TL was assigned as 90 seconds. The mouse was allowed to explore the maze for 10 seconds and then returned to its home cage. Memory retention was examined on the second day, 24 hours after the first day's trial. The animals were treated with EA and standard for 14 days. At the end of treatment period, animals of respective groups are subjected to scopolamine (1mg/kg i.p) treatment, 60 mins after extract administration, except the first group, which served as vehicle control. Transfer latency (TLT) was recorded after 45 minutes of drug administration and after 24 hours.

## Passive avoidance:

The passive avoidance apparatus consisted of a Plexiglas box (30 x 30 x 40 cm) with a steel rod grid floor (29 parallel steel rods, 0.3cm in diameter set 1cm apart). A wooden platform (8 x 8 x 5 cm) was placed in the centre of the grid floor. Intermittent electric shocks (1Hz, 0.5s, 60VDC) were delivered to the grid floor by an isolated stimulator. Each mouse was trained by gently placing it on the platform. When the animal stepped down from the platform and placed all its paws on the grid floor, shock was delivered for 15s [14].

## Statistical analysis:

The results of the study were expressed as mean ± SEM (standard error of mean). The difference between the control and treated means was analyzed using one-way analysis of variance (ANOVA). P-values < 0.05 were taken to be statistically significant. The statistical analysis was done using the software Graph-pad prism version no: 5.0.

## Results:

Table 2 shows the time spent in open arm was 7.6±0.55 sec with GS group mice, 0.89± 0.6 sec with GSP group mice, 5.8±0.84 sec with GSLD group mice and 6.8±0.45 sec with GSHD mice. Statistically significant difference was observed between GS and GSP as well as GSLD group mice, this was also seen between GSP group with low dose and high dose treated mice groups. The time spent in closed arm was 200.8±1.3 sec with GS group mice, 299.4±0.89 sec with GSP group mice, 279.4±5.9 sec with GSLD group mice and 286±4.24 sec with GSHD mice. A statistically significant difference was observed between GS and GSP as well as between GSP and both test dose (GSLD & GSHD) group mice. The time spent in central area was 12±1.58 sec with GS group mice, 25±1.41 sec with GSP group mice, 14.2±1.1 sec with GSLD group mice and 11.4±0.55 sec with GSHD mice. A statistically significant difference was observed between GS and GSP, this was also seen between low dose and high dose treated mice groups. The open arm entries were 2.2±0.45 by GS group mice, 0.4±0.2 by GSP group mice, 1.8±0.84 by GSLD group mice and 2.2±0.45 by GSHD group mice. A significant difference was seen between GS and GSP group mice means. The closed arm entries were 10.6±0.55 by GS group mice, 1.6±0.55 by GSP group mice, 4.6±0.89 by GSLD group mice and 7.6±0.89 by GSHD group mice. A significant difference was seen between GS and GSP group mice means, this was also seen between low dose and high dose treated mice groups. The total transitions were 12.6±0.89 by GS group mice, 3.4±0.55 by GSP group mice, 7±0.71 by GSLD group mice and 10±0.71 by GSHD group mice. A significant difference was seen between GS and GSP group mice means, this was also seen between low dose and high dose treated mice groups. Table 3 shows the mean time in the safe zone, and shock zone for each group of animals. Group 4 spent the least amount of time in the safe zone and the shock zone time. This is when comparing group 2 Vs group 3 and 4 with a statistical significance of p < 0.05.

## Discussion:

The extract significantly increased the time that mice spent exploring a novel environment, which is a measure of spatial memory and found that the extract improved the performance of mice in a passive avoidance test, which is a measure of long-term memory. The mechanisms by which Evolvulus alsinoides exerts its nootropic activity are not fully understood. However, it is thought that the extract may work by increasing the levels of acetylcholine in the brain or by protecting neurons from damage. Our data is supported by Ambika *et al.* explained that the plant-derived compounds are becoming increasingly important in the treatment of a variety of diseases. Many Convolvulaceae plants include chemicals that have been shown to have Nootropic activity, wound-healing and anti-diabetic properties [15]. Mehla *et al.* presented Evolvulus Alsinoides, also known as vishnukranthi, is a commonly used traditional medicine for enhancing memory. Elevated plus maze, passive avoidance and Morris water maze were used for assessment of cognitive function. The results suggest an anti-Alzheimer potential of hydro-alcoholic extract of E. alsinoides [16]. Mehla *et al.* demonstrated that cognitive impairment is a serious health issue linked to ageing, stress, hypertension, and neurodegenerative conditions such as Parkinson's disease and epilepsy [17].

Gomathi *et al.* studied that the accumulation of free radicals in the body causes various oxidative stress-related illnesses. Endogenous systems, exposure to diverse physiochemical situations, and disease states all produce free radicals. For proper physiological function, there must be a balance between free radicals and antioxidants. Many studies are being conducted around the world to discover natural antioxidants derived from plants [18]. Nahata *et al.* characterized that the whole plant of Evolvulus alsinoides Linn has been used professionally for ages in the Ayurvedic School of medicine for its memory-potentiating, anxiolytic, and tranquilizing characteristics. This study aimed to see how Evolvulus alsinoides (EA), affected learning and memory in mice. Nootropic activities, such as Cook and Weidley's pole climbing apparatus, passive avoidance paradigms, and active avoidance tests, were employed to assess learning and memory. In rats, both dosages of all EA extracts increased learning and memory significantly. Furthermore, the amnesia caused by scopolamine was significantly reversed at these levels. In the step-down and shuttle-box avoidance paradigms, nootropic activity was compared using piracetam as the reference, and both showed substantial memory-boosting effects [19]. Sethiya et al. characterized that the Evolvulus alsinoides Linn is an Ayurvedic medication known for its effects on the central nervous system, particularly regarding memory and intelligence. Ayurvedic and other Sanskrit literature revealed the existence of four separate plant species known as Evolvulus alsinoides Linn, which are employed in various Ayurvedic prescriptions documented in ancient books, either alone or in conjunction with other herbs [20].

## Conclusion:

The plant Evolvulus alsinoides has a wide range of potential therapeutic benefits, including nootropic activity. Data shows that Evolvulus alsinoides is a promising candidate for the development of new treatments for memory impairment and other cognitive disorders. However, more data is needed to confirm the safety and efficacy of Evolvulus alsinoides in humans and to investigate its long-term effects.

## Ethical Considerations:

The study was performed in accordance with the universal ethical principles stated in the declaration of Helsinki on human research.

## Code of Ethics:

The study proposal was reviewed and approved by the ethical committee of ACS Medical college and hospital, Dr. MGR educational and research institute (Ethical approval code: VI/IAEC/DrMGR/2053/PO/ReBi/S/19/CPCSEA/28.01.2023/03)

## Authors' contributions:

Study design: KN. Data gathering: KN. Data Analysis: VBC, SR. Drafting the manuscript: PS. Revising the Manuscript: RM. Final Approval: SR, PS, and RM.

## Financial support and sponsorship:

Nil

## Figures and Tables

**Table 1:** Study plan

**Sl. No**	**Groups**	**No. of Mice**	**Drugs (0.5ml)**
I	GS	5	Scopolamine 1mg/kg for 14 days i.p injection
II	GSP	5	Scopolamine 1mg/kg for 14 days i.p injection +Piracetam 50mg/kg 14 days i.p injection
III	GSLD	5	scopolamine (i.p) 45 minutes before giving extract +EA ethanol extract (200 mg/kg, orally, 14 days)
IV	GSHD	5	scopolamine (i.p) 45 minutes before giving extract +EA ethanol extract (400 mg/kg, orally, 14 days)

**Table 2:** Elevated plus maze test of methanolic extract of Evolvulus alsinoides linn (vishnukranthi) in mice with scopolamine-induced amnesia

**Groups**	**Time spent in Open arm (Sec)**	**Time spent in Closed arm (Sec)**	**Time spent in Central platform (Sec)**	**Open arm entries**	**Closed arm entries**	**Total transitions**
1	7.6± 0.55	200.8± 1.3	12± 1.58	2.2± 0.45	10.6± 0.55	12.6± 0.89
2	0.89± 0.6**	299.4± 0.89*	25± 1.41*	0.4± 0.2*	1.6± 0.55**	3.4± 0.55**
3	5.8± 0.84 *	279.4± 5.9*	14.2± 1.1 *	1.8± 0.84*	4.6± 0.59**	7± 0.71**
4	6.8± 0.45*	286± 4.24*	11.4± 0.55*	2.2± 0.45*	7.6± 0.89 **	10± 0.71**
Significance at p < 0.05, 0.001, ANOVA followed by Tukey's multiple comparisons test - *, **

**Table 3:** Passive avoidance test of methanolic extract of Evolvulus alsinoides linn (vishnukranthi) in mice with scopolamine-induced amnesia

**Groups**	**SDL (s) - Step down latency**	
	**Safe zone**	**Shock Zone**
1	123 ± 21.7	8 ± 2.8
2	41.8 ± 20.36	17 ± 5.2
3	34.6 ± 7.06*	21.6 ± 4.9
4	33.6 ± 8.79*	19.6 ± 4.6
*Significance at p < 0.05, ANOVA followed by Tukey's multiple comparisons test
